# The efficacy of biliary and serum macrophage inhibitory cytokine-1 for diagnosing biliary tract cancer

**DOI:** 10.1038/s41598-017-09740-x

**Published:** 2017-08-23

**Authors:** Mitsuru Sugimoto, Tadayuki Takagi, Naoki Konno, Rei Suzuki, Hiroyuki Asama, Ko Watanabe, Jun Nakamura, Yuichi Waragai, Hitomi Kikuchi, Mika Takasumi, Yuki Sato, Takuto Hikichi, Hiromasa Ohira

**Affiliations:** 1 Department of Gastroenterology, Fukushima Medical University, School of Medicine, Fukushima, Japan; 2 Department of Endoscopy, Fukushima Medical University Hospital, Fukushima, Japan

## Abstract

The serum macrophage inhibitory cytokine-1 (MIC-1) levels are elevated in some inflammatory conditions and cancers. We thus compared the levels of biliary and serum MIC-1 and conventional tumour markers between 23 biliary tract cancer (BTC) patients (malignant group) and 29 benign biliary disease patients (benign group) and found that all markers were significantly elevated in the malignant group. The levels of two markers were higher in early BTC (Stage I/II, n = 15) than in the benign group: biliary MIC-1 [12 (0–2153) vs. 678 (0–4429) pg/ml, *P* < 0.01] and serum CA19–9 [13 (2–15,682) vs. 45.1 (2–10,478) U/ml, *P* = 0.02]. A receiver operating characteristic curve analysis revealed that the area under the curve for biliary MIC-1 was greater than that for serum CA19-9 (0.77 vs. 0.73). The cut-off value for biliary MIC-1 in diagnosing early BTC was 581.6 pg/ml, and this value yielded a sensitivity, specificity and accuracy of 71.4%, 82.8%, and 79.1%, respectively. The sensitivity of biliary MIC-1 for diagnosing early BTC was superior to that of biliary cytology (71.4% vs. 8.33%, *P* < 0.01), and the combination of serum MIC-1 with CA19-9 (cut-off value = 4021.2 pg/ml, 42.4 U/ml) was useful for screening BTC (sensitivity = 82.6%, specificity = 72.4%). In conclusion, biliary MIC-1 can effectively diagnose early BTC.

## Introduction

The diagnosis of biliary tract cancer is challenging, and current methods include the evaluation of tumour markers (CEA or CA19-9), biliary fluid cytology, brush cytology, and biopsy. However, the diagnostic ability of these methods is not satisfactory^1–15^. Although CA19-9 has been reported to be elevated in up to 85% of patients with cholangiocarcinoma, CA19-9 elevation can be observed in obstructive jaundice without malignancy. CEA elevation is not observed in obstructive jaundice but occurs in 30% of patients with cholangiocarcinoma^16^.

The efficacy of serum macrophage inhibitory cytokine-1 (MIC-1), which is a type of transforming growth factor-β, for the diagnosis of pancreatic cancer was recently reported^17–19^. Serum MIC-1 has been found to be upregulated by several cancers, inflammation, and cytotoxic drugs^19–21^ and in pancreatic cancer and cholangiocarcinoma patients^17^. Although the relationship between pancreatic cancer and serum MIC-1 has been explored, few studies have examined the relationship between biliary cancer and MIC-1.

Therefore, we investigated the efficacy of serum and biliary MIC-1 for diagnosing biliary tract cancer.

## Results

No significant differences were detected between the benign and malignant groups in terms of age (72.7 ± 10.5 years vs. 71.6 ± 10.6 years, *P* = 0.71) and gender (19 males and 10 females vs. 18 males and 5 females, *P* = 0.37) (Table 1). All markers, including biliary MIC-1 [12 (0–2154) pg/ml vs. 678 (0–6383) pg/ ml, *P* < 0.01], serum MIC-1 [1800 (804–7410) pg/ml vs. 4042 (427–7884) pg/ml, *P* = 0.01], serum CA19–9 [13 (2–15682) U/ml vs. 151.7 (2–19120) U/ml, *P* < 0.01], and serum CEA [1.6 (0.6–5.9) ng/ml vs. 2.6 (0.4–16.8) ng/ml, *P* = 0.04], were significantly lower in the benign group.

**Table 1:** Comparison of each marker between the benign and malignant groups.

	Benign (n = 29)	Malignant (n = 23)	*P* value
Age (years), mean ± SD	72.9 ± 10.7	71.6 ± 10.6	0.71
Gender (Male/Female)	19/10	18/5	0.37
Number of bile samples	29	22	
Number of serum samples	27	22	
Biliary MIC-1 (pg/ml), median (range)	12 (0–2154) (n = 29)	678 (0–6383) (n = 22)	<0.01
Serum MIC-1 (pg/ml), median (range)	1800 (804–7410) (n = 27)	4042 (427–7884) (n = 22)	0.01
Serum CA19-9 (U/ml), median (range)	13 (2–15682) (n = 24)	151.7 (2–19210) (n = 23)	<0.01
Serum CEA (ng/ml), median (range)	1.6 (0.6–5.9) (n = 23)	2.6 (0.4–16.8) (n = 23)	0.04

*SD*, standard deviation; *MIC-1*, macrophage inhibitory cytokine-1.

The comparison of the different marker in the same patient revealed that the area under the curve (AUC) for biliary MIC-1 was greater than that for serum MIC-1 (0.77 vs. 0.70) (Table 2), and the AUC for serum CA19-9 was greater than that for serum MIC-1 (0.78 vs. 0.72). In addition, the AUC for biliary MIC-1 was equal to that for serum CA19-9 (0.78 vs. 0.78).

**Table 2:** Comparison of the different markers in each patient.

	Sensitivity (%)	Specificity (%)	AUC
**Biliary and serum MIC-1 (n = 48, benign 27, malignant 21)**			
Biliary MIC-1	66.7	81.5	0.77
Serum MIC-1	52.4	85.2	0.70
**Serum CA19-9 and Serum MIC-1 (n = 44, benign 22, malignant 22)**			
Serum CA19-9	77.3	68.2	0.78
Serum MIC-1	54.5	86.4	0.72
**Serum CA19-9 and Biliary MIC-1 (n = 46, benign 24, malignant 22)**			
Serum CA19-9	72.7	79.2	0.78
Biliary MIC-1	68.2	83.3	0.78

*AUC*, area under the curve; *MIC-1*, macrophage inhibitory cytokine-1.

**Figure 1:**
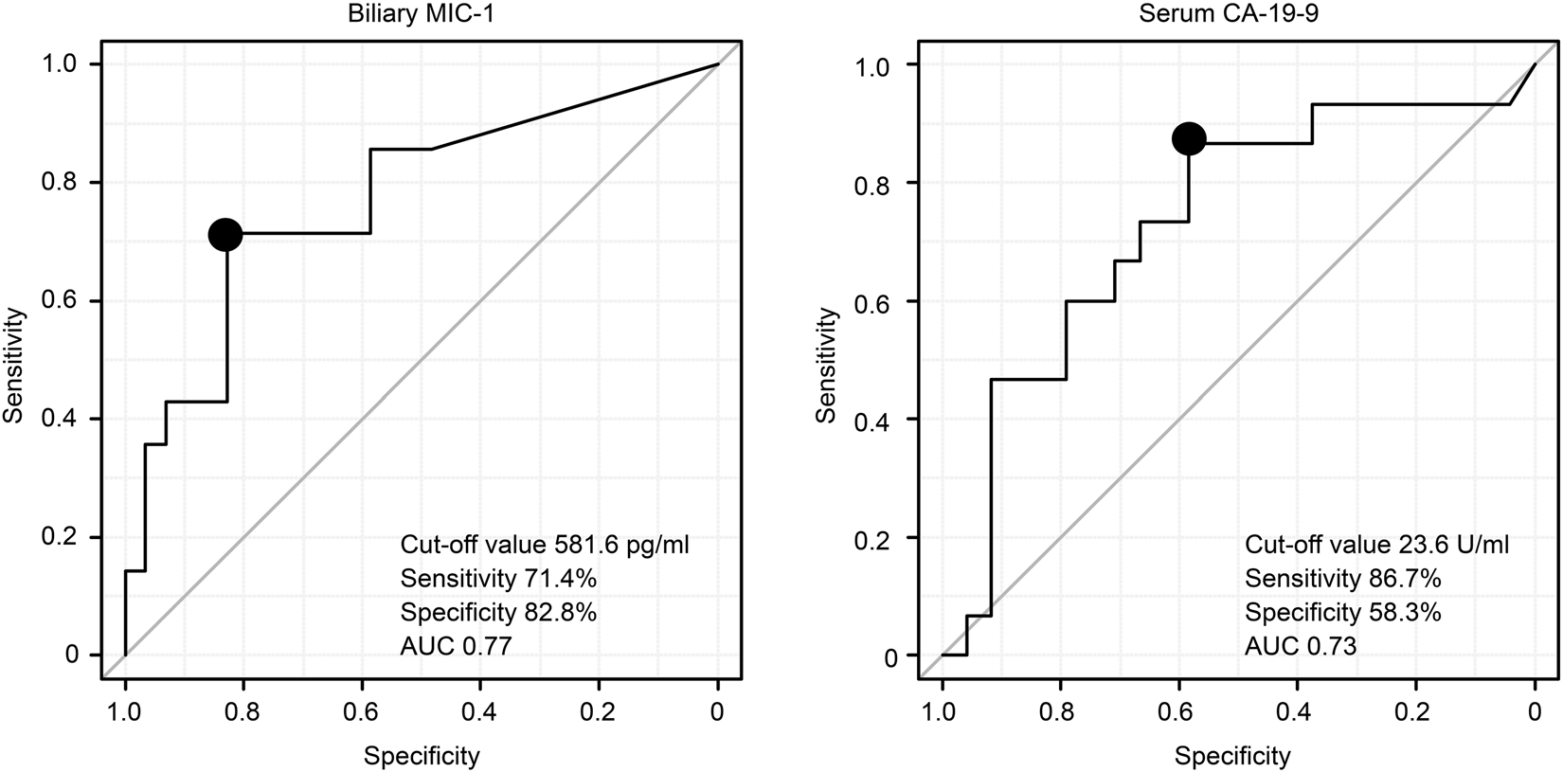
ROC curves of biliary MIC-1 and serum CA 19-9 for the diagnosis of early biliary tract cancer. *ROC*, receiver operating characteristic curve; *MIC-1*, macrophage inhibitory cytokine-1; *AUC*, area under the curve. The cut-off value, specificity, and sensitivity of biliary MIC-1 were 581.6 pg/ml, 71.4%, and 82.8%, respectively. The cut-off value, specificity, and sensitivity of serum CA19-9 were 23.6 pg/ml, 86.7%, and 58.3%, respectively. The AUC for biliary MIC-1 was greater than that for CA19-9 (0.77 vs. 0.73).

No significant differences in serum MIC-1 [1800 (804–7410) pg/ml vs. 2926 (427–6952) pg/ml, *P* = 0.12] and serum CEA [1.6 (0.6–5.9) ng/ml vs. 2.0 (0.4–4.9) ng/ml, *P* = 0.52] were observed between the benign and early malignant groups (Stage I/II, n = 15) (Table 3). Other markers, including biliary MIC-1 [12 (0–2153) pg/ml vs. 678 (0–4429) pg/ml, *P* < 0.01] and serum CA19-9 [13 (2–15682) U/ml vs. 45.1 (2–10478) U/ml, *P* = 0.02], were significantly lower in the benign group than in the early malignant group. The cut-off value of biliary MIC-1 for diagnosing early biliary tract cancer was found to equal 581.6 pg/ml (Fig. 1), and this value showed a sensitivity of 71.4% and a specificity of 82.8%. The cut-off value of serum CA19-9 for diagnosing early biliary tract cancer was found to be 23.6 U/ml, and this cut-off value yielded a sensitivity of 86.7% and a specificity of 58.3%. The AUC for biliary MIC-1 was higher than that for serum CA19-9 (0.77 vs. 0.73).

**Table 3:** Comparison of each marker between the benign and early malignant groups.

	Benign (n = 29)	Malignant Stage I or II (n = 15)	*P* value
Biliary MIC-1 (pg/ml), median (range)	12 (0–2153) (n = 29)	678 (0–4429) (n = 14)	<0.01
Serum MIC-1 (pg/ml), median (range)	1800 (804–7410) (n = 27)	2926 (427–6952) (n = 14)	0.12
Serum CA19-9 (U/ml), median (range)	13 (2–15682) (n = 24)	45.1 (2–10478) (n = 15)	0.02
Serum CEA (ng/ml), median (range)	1.6 (0.6–5.9) (n = 23)	2.0 (0.4–4.9) (n = 15)	0.52

*MIC-1*, macrophage inhibitory cytokine-1.

The sensitivity of biliary MIC-1 for early malignant diagnosis was significantly higher than that of biliary cytology [71.4% (10/14) vs. 8.33% (1/12), *P* < 0.01] (Table 4), but the specificity of biliary MIC-1 was lower than that of biliary cytology [82.8% (24/29) vs. 100% (14/14), *P* = 0.156]. The accuracy of biliary MIC-1 for early malignant diagnosis was higher for than that of biliary cytology [79.0% (34/43) vs. 57.6% (15/26), *P* = 0.099].

**Table 4:** Comparison of early malignant diagnosability between biliary MIC-1 and biliary cytology.

	Biliary MIC-1	Biliary cytology	*P* value
Sensitivity (%)	71.4 (10/14)	8.33 (1/12)	<0.01
Specificity (%)	82.8 (24/29)	100 (14/14)	0.16
Accuracy (%)	79.0 (34/43)	57.6 (15/26)	0.10

*MIC-1*, macrophage inhibitory cytokine-1.

## Discussion

In this study, we investigated the efficacy of biliary and serum MIC-1 for the diagnosis of malignant biliary diseases and found that biliary MIC-1 was significantly elevated in early biliary tract cancer patients. Additionally, biliary MIC-1 is more effective than other markers and biliary cytology for the diagnosis of early biliary tract cancer.

Biliary MIC-1 was significantly elevated in the biliary tract cancer (and the Stage I/II biliary tract cancer) group compared with the benign group. However, biliary MIC-1 is not suitable for screening because endoscopic retrograde cholangiopancreatography (ERCP) or percutaneous transhepatic gallbladder drainage (PTGBD) must be performed for bile collection. However, according to the receiver operating characteristic (ROC) curve analysis, the sensitivity of the serum MIC-1 cut-off value was found to equal 54.5% (12/22) (Table 5), but this value is too low to be used as a cut-off value for screening biliary tract cancer. Therefore, we used the combination of serum MIC-1 (cut-off value of 4021.2 pg/ml, as determined through a ROC curve analysis) with serum CA19-9 (cut-off value of 42.4 U/ml, as determined through a ROC curve analysis) (Table 5). In past studies regarding the diagnosis of pancreatic cancer, the combination of serum MIC-1 and serum CA19-9 levels was found to be more effective than the use of serum MIC-1 or serum CA19-9 alone^17, 18^. In this study, the combination of serum MIC-1 with CA19-9 yielded a sensitivity of 82.6% (19/23) and a specificity of 72.4% (21/29), which are sufficiently high for screening biliary tract cancer.

**Table 5:** Diagnosis of biliary tract cancer by the combination of serum MIC-1 and serum CA19-9.

	Cut-off value	Sensitivity (%)	Specificity (%)
Serum MIC-1	4021 pg/ml	54.5 (12/22)	85.2 (23/27)
Serum CA19-9	42.4 U/ml	73.9 (17/23)	79.2 (19/24)
Combination of serum MIC-1 and CA19-9		82.6 (19/23)	72.4 (21/29)

*MIC-1*, macrophage inhibitory cytokine-1.

Biliary MIC-1 was identified as the most effective marker for the diagnosis of early biliary tract cancer in this study. With the exception of surgery, biliary cytology and biopsy are the only methods currently available to determine malignancy in biliary tract diseases. As mentioned previously, the ability of biliary cytology or biliary brush cytology to diagnose malignant disease has been reported to be unsatisfactory^1–3, 5–7, 10, 12–14^. In fact, only one of 12 early biliary tract cancer patients included in this study was diagnosed with malignancy by biliary cytology. Biliary biopsy has been reported to be equivalent or superior to biliary brush cytology for the diagnosis of biliary cancer^2, 6, 7, 13^. In fact, 12 of the patients included in this study who did not exhibit malignancy by biliary cytology were diagnosed with biliary cancer by biliary biopsy. However, two patients who did not exhibit malignancy by biliary biopsy showed an MIC-1 level that was higher than the cut-off value, which shows that the measurement of biliary MIC-1 contributes to the diagnosis of biliary tract cancer.

Furthermore, the serum MIC-1 level has been reported to be elevated in Stage I/II pancreatic cancer^19^. This study found that patients who showed biliary MIC-1 levels higher than the cut-off value were significantly more likely to belong to the Stage I/II biliary cancer group than the benign group, which demonstrates that biliary MIC-1 might be useful for diagnosing biliary tract cancer in the early stages.

This study has several limitations. First, the study included a small number of patients at one institution, and we hope that a larger study will be performed in the future. Second, the serum MIC-1 level has been reported to be elevated in other malignant diseases^17, 19, 21, 22^, and higher values have been observed in pancreatic cancer and cholangiocarcinoma^17, 19^. We measured the biliary and serum MIC-1 levels of 16 pancreatic cancer patients. The biliary MIC-1 level was found to be equivalent in biliary cancer and pancreatic cancer [678 (0–6383) pg/ml vs. 1031 (0–5937) pg/ml, *P* = 0.99] according to the Mann-Whitney U test, and the serum MIC-1 levels were also found to be equivalent (3860 ± 2271 pg/ml vs. 3272 ± 1438 pg/ml, *P* = 0.36) according to Student’s t test.

In conclusion, the use of the serum MIC-1 and serum CA19-9 levels is effective for screening biliary tract cancer, and the biliary MIC-1 level is more effective for diagnosing early biliary tract cancer than conventional serum tumour markers and biliary cytology.

## Methods

### Study design

We measured the biliary and serum MIC-1 levels of patients with biliary tract diseases and their efficacy for the diagnosis of biliary tract cancer compared with the serum CA19-9 or CEA levels or biliary cytology. This study was approved by the ethics committee of Fukushima Medical University. The methods were performed in accordance with the approved guidelines.

### Patients

We recruited 52 biliary tract disease patients, and biliary fluid and blood from these patients were collected between May 2015 and March 2017. Thirty-three of these patients were diagnosed with malignant biliary tract cancer. Among them, 22 patients had biliary ductal cancer, and one patient had gallbladder cancer. The other 29 patients were diagnosed with benign biliary tract diseases or biliary stricture of unknown origin (central biliary ductal stones, 19; chronic pancreatitis, 2; autoimmune pancreatitis, 2; cholecystitis, 1; acute pancreatitis, 1; Lemmel syndrome, 1; primary sclerosing cholangitis, 1; exclusion by intraductal papillary neoplasm, 1; and central biliary duct stricture of unknown origin, 1) (Table 6). The patients provided written informed consent.

**Table 6:** Biliary tract diseases or origin of benign biliary stricture in targeted patients.

Benign (n = 29)		Malignant (n = 23)	
CBD stones	19	Biliary ductal cancer	22
Chronic pancreatitis	2	Gall bladder cancer	1
Autoimmune pancreatitis	2		
Acute pancreatitis	1		
Cholecystitis	1		
Lemmel syndrome	1		
PSC	1		
IPMN	1		
CBD stricture by unknown origin	1		

*CBD*, central biliary duct; *PSC*, primary sclerosing cholangitis; *IPMN*, intraductal papillary neoplasm.

### Collection of bile and preservation of bile and serum

Bile samples were collected by ERCP or PTGBD.

The ERCP procedures were performed as follows. Before the ERCP procedure was initiated, all patients were sufficiently sedated with midazolam. After the ERCP endoscope was placed in the descending portion of the duodenum, biliary duct cannulation was performed, and bile was collected into centrifuge tubes. The bile samples were frozen at −80 °C. For the PTGBD procedure, the patients were anaesthetized with pentazocine. After the PTGBD tube was inserted, a bile sample was collected and frozen at −80 °C as described above. The serum samples were preserved at −80 °C.

### Diagnosis of malignancy

Patients were diagnosed with malignancy through bile cytology, biliary biopsy, endoscopic ultrasonography-guided fine needle aspiration, surgery, or positron emission tomography. Class IV/V cases were treated as malignant cases.

### Measurement of serum and biliary MIC-1

Frozen serum and bile were thawed at room temperature. The bile samples were centrifuged for 10 minutes at 13,000 rpm, and the MIC-1 levels were then measured using a Quantikine ELISA Human GDF-15 immunoassay kit (R&D systems, Minneapolis, MN, United States) according to the manufacturer’s instructions.

### Examined items

The patient characteristics (age, gender) as well as the biliary and serum MIC-1, the serum CA19-9 and the serum CEA levels were compared between the benign and malignant groups. Comparisons of malignant diagnosability between serum MIC-1 and biliary MIC-1, serum CA19-9 and serum MIC-1, and serum CA19-9 and biliary MIC-1 of the same patients were performed. Additionally, we compared these markers between the benign and early malignant group (Stage I/II biliary tract cancer, based on the UICC classification). The ability to diagnose malignancy and the cut-off values of these markers were compared between the benign and malignant groups. Finally, the diagnostic ability of the most effective marker was compared with that of bile cytology.

### Statistical analyses

Student’s t test or the Mann-Whitney U test was used to compare age, serum and biliary MIC-1 levels, and serum CA19-9 and CEA levels between the benign and malignant groups. Fisher’s exact test was used to compare the gender distribution between the benign and malignant groups. ROC curves were used to compare the ability to diagnose for malignancy of several markers that exhibited significantly different levels between the benign and malignant groups. A *P* value < 0.05 was considered statistically significant in all statistical analyses. All statistical analyses were performed using the EZR platform (Saitama Medical Centre, Jichi Medical University, Saitama, Japan), which is a graphical user interface for R (The R Foundation for Statistical Computing, Vienna, Austria). More precisely, EZR is a modified version of the R commander designed to perform functions that are frequently used in biostatistics^23^.
